# Meta-analysis of the impact of alpha-glucosidase inhibitors on incident diabetes and cardiovascular outcomes

**DOI:** 10.1186/s12933-019-0933-y

**Published:** 2019-10-17

**Authors:** Ruth L. Coleman, Charles A. B. Scott, Zhihui Lang, M. Angelyn Bethel, Jaakko Tuomilehto, Rury R. Holman

**Affiliations:** 1 Diabetes Trials Unit, OCDEM, University of Oxford, Churchill Hospital, Old Road, Headington, Oxford, OX3 7LJ UK; 2 Bayer Healthcare Company Ltd, Beijing, China; 3 Department of Public Health Solutions, National Institute for Health and Welfare, 00271 Helsinki, Finland; 4 Department of Public Health, University of Helsinki, 00014 Helsinki, Finland; 5 Saudi Diabetes Research Group, King Abdulaziz University, Jeddah, 21589 Saudi Arabia

**Keywords:** Impaired glucose tolerance, Type 2 diabetes, Cardiovascular disease, Alpha glucosidase inhibitor, Meta-analysis

## Abstract

**Background:**

Alpha-glucosidase inhibitors (AGIs) have been shown to reduce incident type 2 diabetes but their impact on cardiovascular (CV) disease remains controversial. We sought to identify the overall impact of AGIs with respect to incident type 2 diabetes in individuals with impaired glucose tolerance (IGT), and CV outcomes in those with IGT or type 2 diabetes.

**Methods:**

We used PubMed and SCOPUS to identify randomized controlled trials reporting the incidence of type 2 diabetes and/or CV outcomes that had compared AGIs with placebo in populations with IGT or type 2 diabetes, with or without established CV disease. Eligible studies were required to have ≥ 500 participants and/or ≥ 100 endpoints of interest. Meta-analyses of available trial data were performed using random effects models to calculate hazard ratios (HRs) and 95% confidence intervals (CIs) for incident type 2 diabetes and CV outcomes.

**Results:**

Of ten trials identified, three met our inclusion criteria for incident type 2 diabetes and four were eligible for CV outcomes. The overall HR (95% CI) comparing AGI with placebo for incident type 2 diabetes was 0.77 (0.67–0.88), p < 0.0001, and for CV outcomes was 0.98 (0.89–1.10), p = 0.85. There was little to no heterogeneity between studies, with I^2^ values of 0.03% (p = 0.43) and 0% (p = 0.79) for the two outcomes respectively.

**Conclusions:**

Allocation of people with IGT to an AGI significantly reduced their risk of incident type 2 diabetes by 23%, whereas in those with IGT or type 2 diabetes the impact on CV outcomes was neutral.

## Background

Alpha-glucosidase inhibitors (AGIs) such as acarbose, miglitol and voglibose are oral drugs used in the management of diabetes, primarily to reduce post-prandial glucose concentrations. Their use in individuals with impaired glucose tolerance (IGT) has been shown to delay progression to diabetes, but their effects on cardiovascular (CV) outcomes remain uncertain [1]. A previous systematic review evaluated the effects of acarbose on various outcomes, including CV, but it did not include a meta-analysis of randomized controlled trials [2].

STOP-NIDDM was a placebo-controlled randomized trial conducted in centres across Canada, Germany, Austria, Norway, Denmark, Sweden, Finland, Israel, and Spain that randomly allocated participants with IGT to 100 mg acarbose or placebo three times daily [3]. The trial showed a 25% reduction in the incidence of type 2 diabetes with acarbose, compared with placebo (hazard ratio (HR) 0.75, 95% confidence interval (CI) 0.63–0.90, p = 0.001) over a mean follow-up of 3.3 years. A pre-specified secondary analysis, also showed that there was a 49% reduction in cardiovascular outcomes over the same time period (HR 0.51 [0.28–0.95], p = 0.03) suggesting that acarbose might confer cardio protection, although this finding was based on only 47 CV events in total. Similarly, a meta-analysis of seven studies conducted between 1987 and 1999 reported a reduction in myocardial infarction (MI) in individuals assigned acarbose compared with placebo, but was based on only 28 events [4]. When a composite CV outcome (MI, stroke, CV death, angina or coronary revascularization) was examined, a 35% reduction in events was demonstrated based on 167 events in total [4].

The recently completed Acarbose Cardiovascular Evaluation (ACE) trial [5] was a randomized, double-blind, placebo-controlled, phase 4 study conducted in China that recruited patients with coronary heart disease and IGT. Participants were randomly allocated to acarbose (50 mg three times daily) or placebo, given in addition to fully-optimized CV secondary prevention therapy. The ACE trial showed that acarbose delayed progression to type 2 diabetes by 18% (odds ratio 0.82 (95% CI 0.71–0.94, p = 0.005) but was neutral with respect to major adverse cardiovascular events (HR 0.98, 0.86–1.11, p = 0.73).

The aim of this analysis was to identify the overall impact of AGIs with respect to incident type 2 diabetes specifically in individuals with IGT, and on combined CV outcomes in those with IGT or type 2 diabetes.

## Methods

### Data sources and searches

We conducted a meta-analysis of randomized placebo-controlled trials of AGIs in populations with IGT or type 2 diabetes, with or without established CV disease. Guidelines published by the Cochrane Collaboration for the conduct of a meta-analysis and the PRISMA checklist [6] for reporting were followed to ensure best practice. This meta-analysis was registered with PROSPERO, an international prospective register of systematic reviews. PROSPERO registration provides transparency in the review process and helps counter publication bias by providing a permanent record of prospectively registered reviews, whether they are eventually published or not. Our PROSPERO submission included publication of key information relating to the design and conduct of the meta-analysis [7].

Two reviewers (RLC, CABS) independently screened titles/abstracts and full texts for eligibility, assessed risk of bias, and collected data from each eligible study. Any reviewer disagreements were resolved by discussion. Ethics approval and patient consent were not required for these analyses.

We used PubMed and SCOPUS to conduct literature searches to identify relevant studies, with no language restrictions for trials from inception up to the 28th of February 2018. An initial search of published systematic reviews/meta-analyses concerning AGIs was performed to identify commonly used terms relating to AGIs. Pre-defined search terms (plus spelling variations) included “alphaglucosidase inhibitor”, “acarbose”, “voglibose”, “miglitol”, “cardiovascular outcomes”, “type 2 diabetes”, “impaired glucose tolerance”, “postprandial hyperglycaemia”, “dysglycemia” and “randomized controlled trials”. For these analyses, only published articles were considered.

### Study selection

The search results were filtered to include only those randomized controlled trials comparing an AGI with placebo on progression to type 2 diabetes and/or CV outcomes in individuals with IGT or type 2 diabetes, with or without a history of CV disease. To avoid the inclusion of short-term small-scale trials with little or no outcome data, we specified in advance that those selected should have a ≥ 500 human participants and/or ≥ 100 pre-defined cardiovascular/diabetes events, with at least 1 year of follow up. Trials reporting CV outcomes were required to include as a minimum all three components of a major adverse cardiovascular event (MACE-3) composite outcome defined as CV death, non-fatal MI or non-fatal stroke. Trials reporting diabetes outcomes were required to specify type 2 diabetes diagnosed by two successive glucose values (fasting plasma glucose [FPG] ≥ 7.0 mmol/l or 2-h post challenge plasma glucose [2hrPG] ≥ 11.1 mmol/l).

### Data extraction and quality assessment

Sources of reporting bias, e.g*.* publication bias, language bias, citation bias, were examined using funnel plots.

### Data synthesis and analysis

Study-level data for all eligible trials identified were extracted from their corresponding published papers. We performed a random effects meta-analysis, with each study weighted according to the inverse variance method. HRs and 95% CIs were calculated for both incident diabetes and CV outcomes. Possible heterogeneity between studies was examined using Cochrane’s Q-test and the I^2^ inconsistency index used to quantify the percentage of total variation across all the studies. A Q test P-value of < 0.05 indicates significant heterogeneity. I^2^ heterogeneity thresholds are defined as low (≤ 25%), moderate (26–49%) or high (≥ 50%) [8]. The same statistical methodologies were applied separately to incident diabetes and to CV outcomes. All analyses were performed using SAS version 9.4 and/or R version 3.4.0 [9].

## Results

### Incident diabetes

**Fig. 1:**
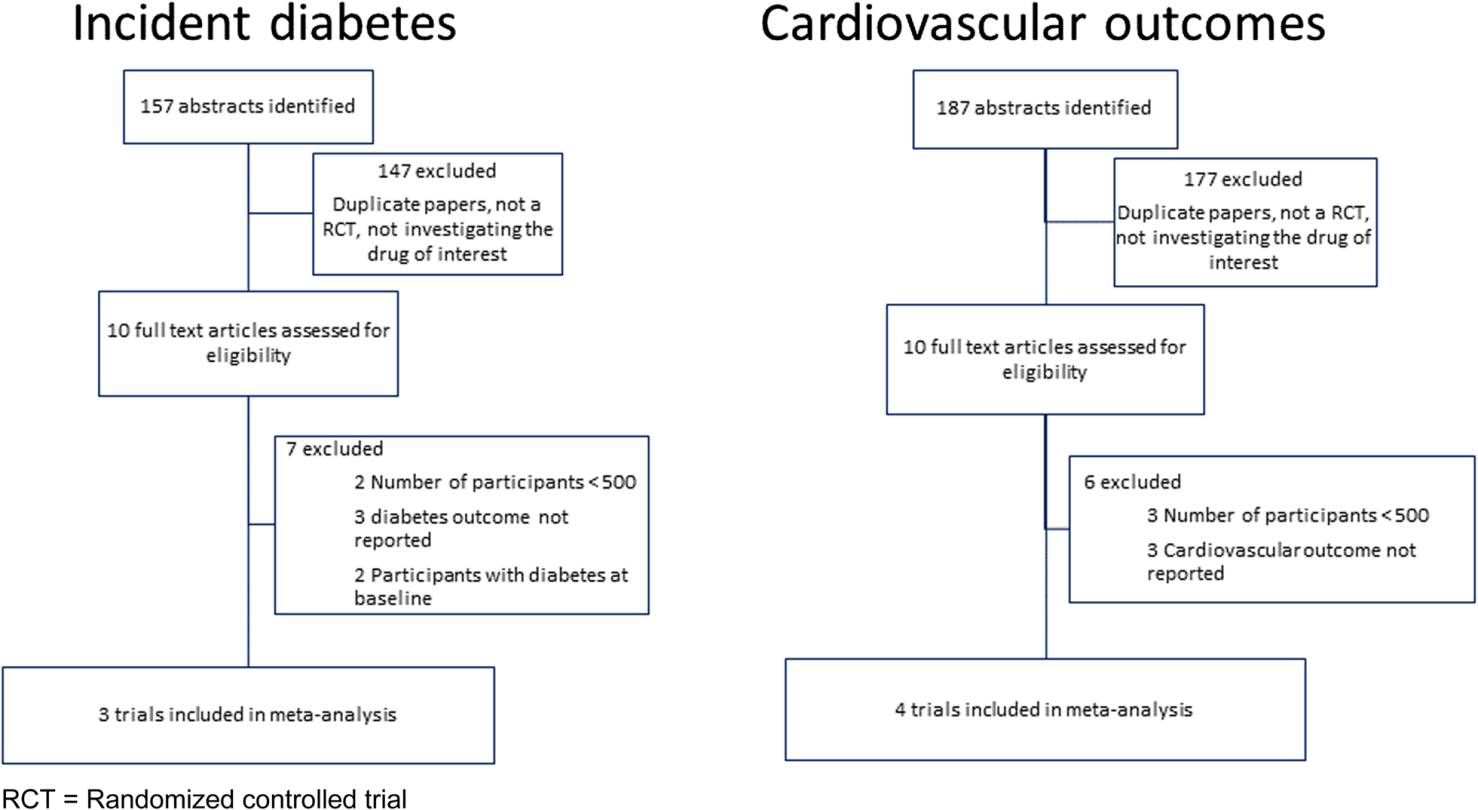
Selection of eligible trials

**Fig. 2:**
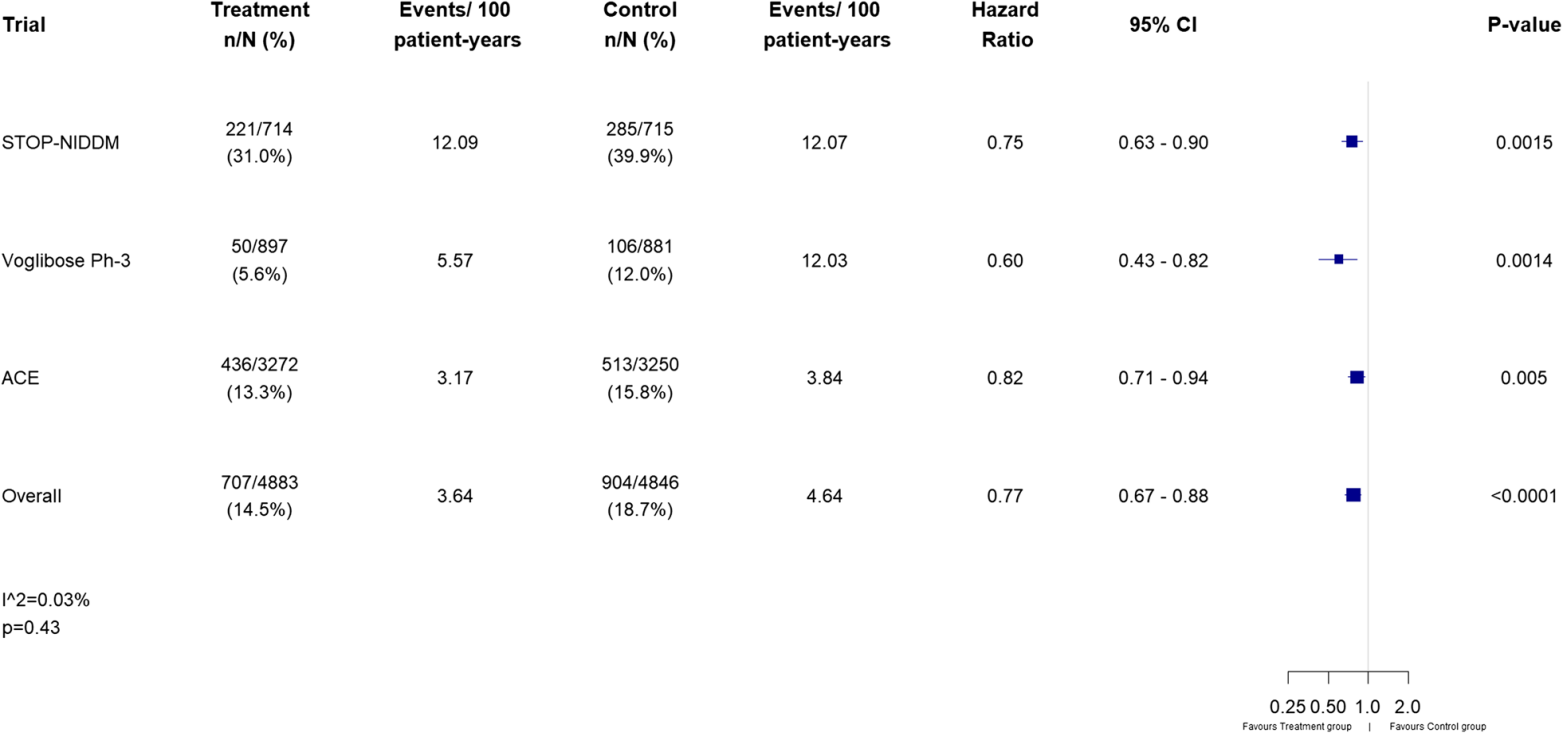
Forest plot of incident diabetes results for each trial and overall effect

Our literature search identified 157 articles, of which there were ten trials that met the criteria for possible inclusion in the incident diabetes meta-analysis (Fig. 1), but only three remained eligible after detailed review. These were the Study to Prevent Non-Insulin-Dependent Diabetes Mellitus (STOP-NIDDM), [3] the voglibose Ph-3 Study, [10] and the ACE trial [5] (Table 1). Participants in these trials were at high risk of developing type 2 diabetes, but people experiencing a CV event in the pre-screening period (3 months for ACE, 6 months for the other 2 studies) were excluded. All three trials reported statistically significant relative risk reductions for incident diabetes in the treatment group, compared with the placebo group, of 25%, 40% and 18% for STOP-NIDDM, voglibose Ph-3 and ACE respectively. This equated to an overall 23% reduction for AGIs (HR 0.77, 95% CI 0.67–0.88, p < 0.0001), with an I^2^ value of 0.03% (p = 0.43) suggesting minimal heterogeneity between studies (Fig. 2), and the funnel plot (Additional file 1: Figure S1) indicating that publication bias is unlikely.

**Table 1:** Characteristics of trials included in meta-analyses

	STOP-NIDDM (n = 1429)		Voglibose Ph-3 (n = 1778)		ABC (n = 859)		ACE (n = 6522)		UKPDS (n = 1946)	
Year published	2002		2009		2017		2017		1998	
Recruitment dates	December 1995–July 1998		October 2003–March 2007		May 2005–June 2012		March 2009–October 2015		May 1994–September 1994	
Dose	100 mg three times daily		0.2 mg three times daily		0.2 mg three times daily		50 mg three times daily		100 mg three times daily	
Inclusion criteria	Impaired glucose tolerance		Impaired glucose tolerance		Impaired glucose tolerance and previous MI		Impaired glucose tolerance and established coronary heart disease		N/A	
Cardiovascular outcome	Coronary heart disease^a^, CVD death, congestive heart failure, cerebrovascular events and peripheral vascular disease		N/A		CVD composite endpoint^b^		MACE- 5^c^		MACE-5^c^	
Diabetes outcome	New-onset diabetes		New-onset diabetes		N/A		New-onset diabetes		N/A	

	Acarbose (n = 714)	Placebo (n = 715)	Voglibose (n = 897)	Placebo (n = 881)	Voglibose (n = 424)	Placebo (n = 435)	Acarbose (n = 3272)	Placebo (n = 3250)	Acarbose (n = 973)	Placebo (n = 973)
Age (years)	54.3 ± 7.9	54.6 ± 7.9	55.7 ± 9.1	55.7 ± 9.2	67 (57, 73)	65 (58, 73)	64.4 ± 8.2	64.3 ± 8.0	60 ± 9	60 ± 9
BMI (Kg/m^2^)	31.0 ± 4.3	30.9 ± 4.2	25.8 ± 3.7	25.9 ± 3.8	24.2 (22.5, 26.0)	24.3 (22.5,26.3)	25.3 ± 3.1	25.5 ± 3.1	29.8 ± 5.6	29.6 ± 5.7
Female	353 (52%)	342 (50%)	356 (40%)	351 (40%)	60 (14.2%)	58 (13.3%)	877 (27%)	885 (27%)	369 (37.9%)	344 (35.4%)

Data are mean ± 1 SD or median (IQR)

^a^Myocardial infarction, new angina, revascularization procedures

^b^CV death, non-fatal MI, Non-fatal unstable angina, non-fatal stroke, coronary revascularization procedure

^c^CV-death, non-fatal stroke, non-fatal MI, hospitalization for unstable angina and hospitalization for heart failure

NB: Diabetes was diagnosed when two successive diagnostic plasma glucose values of either FPG ≥ 7·0 mmol/L or 2hrPG ≥ 11·1 mmol/L was obtained

### Cardiovascular outcomes

Of the 187 articles identified in the literature search, there were ten trials that met the criteria for possible inclusion in the CV outcomes meta-analysis (Fig. 1), but only four of these remained eligible after detailed review. These were the UK Prospective Diabetes Study (UKPDS), [11] STOP-NIDDM, [3] the Alpha-glucosidase-inhibitor Blocks Cardiac Events in Patients with Myocardial Infarction and Impaired Glucose Tolerance (ABC) trial, [12] and the ACE trial [5] (Table 1).

**Fig. 3:**
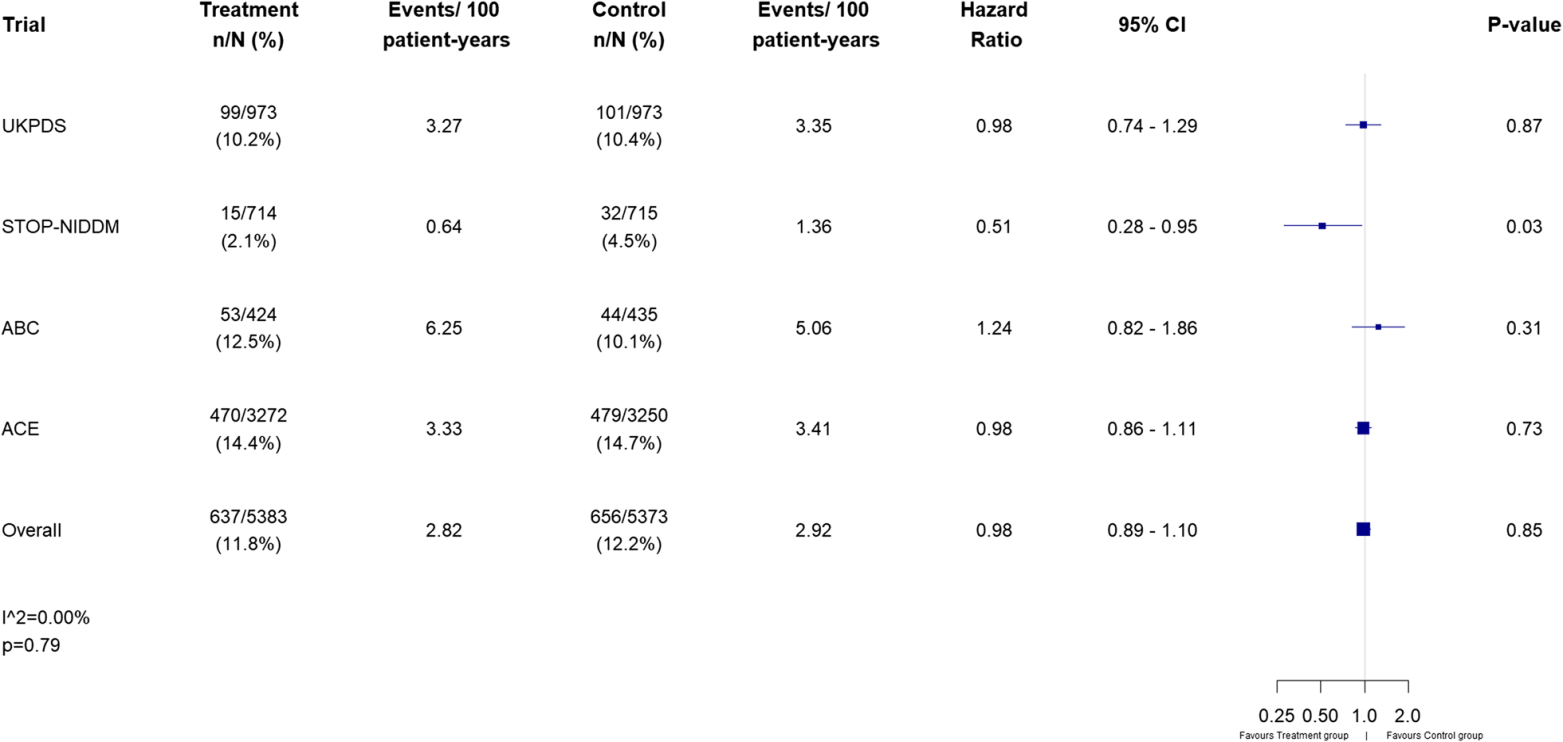
Forest plot of incident cardiovascular outcome results for each trial and overall effect

The CV outcome definition used in all four trials was MACE-3: plus unstable angina and congestive heart failure for UKPDS and ACE; plus angina, coronary revascularization, congestive heart failure and peripheral vascular disease for STOP-NIDDM; plus unstable angina and coronary revascularization for ABC. UKPDS, ABC and ACE showed no difference between treatment groups with respect to their CV outcome, with HRs ranging from 0.98 to 1.24 for AGI compared with placebo). STOP-NIDDM, however, showed a 49% reduction (HR 0.51, 95% CI 0.28–0.95, p = 0.03) but with only 47 composite CV events in total, compared with 100 in UKPDS and 949 in ACE. Overall, our meta-analysis showed no reduction in CV events for AGIs compared with placebo (HR 0.98, 95% CI 0.89–1.10, p = 0.85), with an I^2^ value of 0% (p = 0.79) showing no heterogeneity between studies (Fig. 3), and the funnel plot (Additional file 1: Figure S2) indicating that publication bias is unlikely.

## Discussion

This meta-analysis shows an overall statistically significant 23% reduced risk of new-onset diabetes with AGIs in people with IGT (p < 0.0001). These results affirm the benefit of AGIs in reducing the risk of new-onset diabetes, and with acarbose licensed in China and 52 other countries for the treatment of IGT affords a pharmacological opportunity to help in the battle to contain the worldwide diabetes epidemic of type 2 diabetes [13].

AGIs, however, showed no overall impact of on CV outcomes, suggesting that the 49% risk reduction reported for the STOP-NIDDM [3] was most likely a chance finding, particularly with so few events for this secondary outcome, although this was a primary CVD prevention population mostly not taking cardioprotective medications such as statins and renin-angiotensin system inhibitors. An earlier meta-analysis of randomised controlled trials of alpha-glucosidase inhibitors suggested that they may prevent the progression if carotid intima thickness in patients with IGT or type 2 diabetes, [14] but our CV meta-analysis suggests this does not translate into fewer CV events. Our analysis was driven by the neutral outcome results reported by ACE [5] and UKPDS [11], with 949 and 200 events respectively, suggests that AGIs neither increase nor decrease the risk of major adverse cardiovascular events in people with IGT or diabetes, although given the mean follow-up for this analysis is 4.0 years the possibility that AGI use might reduce CV risk in the longer term cannot be excluded. Aggressive treatment of other cardiovascular risk factors, such as hypertension and dyslipidemia, as well as the use of antiplatelet therapy and inhibitors of the renin angiotensin system have resulted in significant reductions in CVD events in populations with [15] or without diabetes [16]. However, the impact of glycaemic reduction on CV disease is modest, [17] possibly making it difficult to detect the effect of some glucose-lowering agents, especially when added to optimized CV risk therapy. Recent studies have demonstrated cardio protection with sodium-glucose cotransporter-2 inhibitors and glucagon-like peptide-1 receptor agonists [18, 19] probably via non-glycaemic mechanisms. AGIs, however, might reduce CV risk indirectly in the longer term by delaying the onset of type 2 diabetes in people with IGT [20]. Such an effect was seen in the Chinese Da Qing study where a 6-year lifestyle intervention program which delayed the onset of type 2 diabetes was shown to be associated with an 11.9% reduction in CV death and a 28.1% reduction in all-cause mortality after 23 years follow-up [21].

Strengths of these analyses include the use of data from all randomized controlled trials reporting outcomes that could be compared, with a minimum 1-year follow-up to enable collection and adjudication of the required endpoints (>1600 incident diabetes events and > 1200 CV events). Our meta-analysis has several limitations. We used study-level rather than patient-level data, which is considered the gold standard for meta-analysis and which restricts our ability to investigate further any subgroups of interest.

Only a small number of eligible trials could be identified, although it is noteworthy that for CV outcomes no other trials with published CV outcomes would have been excluded by our selection criteria. For incident diabetes, a further two small-scale trials were identified but not included. These were the Early Diabetes Intervention programme (EDIP), which reported 62 events of progression to type 2 diabetes defined as FPG ≥ 7.8 mmol/l in 196 subjects followed for 5 years, [22] and the Dutch acarbose intervention study in persons with impaired glucose tolerance (DAISI) which reported 25 events of progression to diabetes in 118 individuals followed for 3 years based on a single glucose measurement [23]. Although these trials did not meet our inclusion criteria, we conducted a sensitivity analysis for incident diabetes which yielded a similar result (HR 0.74 [95% CI 0.67–0.82], p < 0.0001). In addition, the Cochrane collaboration recently published the results of trials reporting the incidence of type 2 diabetes, and separately CV mortality, non-fatal MI and non-fatal stroke in all populations without normal glucose levels (impaired fasting glucose, IGT or elevated HbA_1c_) [24], which yielded similar results to our analysis showing a type 2 diabetes incidence risk ratio of 0.73 (95% CI 0.59–0.90), but the combined MACE-3 endpoint was not reported.

## Conclusion

This meta-analysis demonstrates that the overall impact of AGIs on CV outcomes is neutral, it is clear that they cannot be indicated for CV secondary prevention. To date, although many countries have licensed AGIs for use in IGT, very few currently approve any medication for diabetes prevention. Given that this meta-analysis demonstrates that allocation of people with IGT to an AGI can significantly reduce their risk of incident diabetes, AGIs should be considered as one approach to delaying or preventing new-onset diabetes in people with or without pre-existing CV disease.

## Supplementary information

**Additional file 1: Figure S1.** Funnel plot of included incident diabetes trials. **Figure S2.** Funnel plot of included cardiovascular outcome trials.

## Data Availability

Not applicable.

## Abbreviations

AGI: alpha glucosidase inhibitor
IGT: impaired glucose tolerance
CV: cardiovascular
HR: hazard ratio
CI: confidence interval
MI: myocardial infarction
ACE: acarbose cardiovascular evaluation
MACE: major adverse cardiovascular event
FPG: fasting plasma glucose
2hrPG: two-hour post challenge plasma glucose
STOP-NIDDM: study to Prevent Non-Insulin-Dependent Diabetes Mellitus
UKPDS: uK Prospective Diabetes Study
ABC: alpha-glucosidase-inhibitor Blocks Cardiac Events in Patients with Myocardial Infarction and Impaired Glucose Tolerance
