# Supplementary material for: Meta-analysis of the impact of alpha-glucosidase inhibitors on incident diabetes and cardiovascular outcomes
Source: Cardiovasc Diabetol. 2019 Oct 17;18:135. doi: 10.1186/s12933-019-0933-y (PMC6798440; doi:10.1186/s12933-019-0933-y)
Supplement: Supplementary file 1 — Additional file 1: Figure S1. Funnel plot of included incident diabetes trials. Figure S2. Funnel plot of included cardiovascular outcome trials. [file 12933_2019_933_MOESM1_ESM.docx]

**Additional file Appendix**

**Meta-analysis of the impact of alpha-glucosidase inhibitors on incident diabetes and cardiovascular outcomes**

**Figure S1**

**
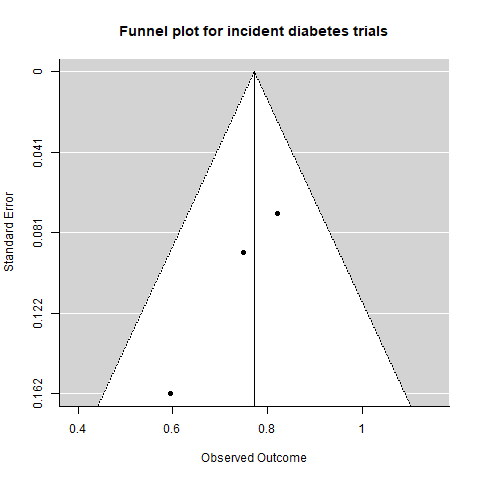
**

**Figure S2**

**
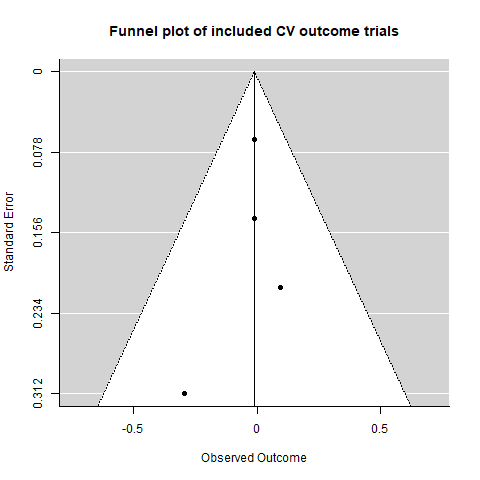
**
